# Comparison of clinical outcomes between unilateral biportal endoscopic discectomy and percutaneous endoscopic interlaminar discectomy for migrated lumbar disc herniation at lower lumbar spine: a retrospective controlled study

**DOI:** 10.1186/s13018-023-04484-z

**Published:** 2024-01-03

**Authors:** Shan Wu, Dian Zhong, Guosheng Zhao, Yang Liu, Yang Wang

**Affiliations:** Department of Spine Surgery, The Second Affiliated Hospital of Chongqing Medical University, No.76, Linjiang Road, Yuzhong District, Chongqing, 400010 China

**Keywords:** Unilateral biportal endoscopic discectomy, Percutaneous endoscopic interlaminar discectomy, Migrated lumbar disc herniation

## Abstract

**Background:**

Both Unilateral Biportal Endoscopic Discectomy (UBED) and Percutaneous Endoscopic Interlaminar Discectomy (PEID) have resulted in favorable clinical outcomes in the management of LDH. The aim of this study is to comprehensively compare the efficacy of UBED and PEID in treating migrated LDH in the lower lumbar spine, with a specific focus on high-grade migrated LDH.

**Methods:**

96 patients who underwent UBED (31 cases) and PEID (65 cases) procedures were enrolled in the study. All patients received a minimum follow-up period of 6 months. Clinical outcomes of the patients were assessed with incision length, operation time, total hemoglobin loss, hospital stay, intraoperative fluoroscopy times, visual analogue scale (VAS) for lower back and leg pain, Oswestry disability index (ODI), modified MacNab criteria, complications, area of lamina loss and increased intervertebral height.

**Results:**

The VAS scores for lower back and leg pain and ODI significantly decreased in both groups after the operation. Preoperatively, at 1 day, 1 month, and 6 months after the procedure, the VAS and ODI scores exhibited no significant differences between the two groups. There was no significant difference in terms of modified MacNab criteria, area of lamina loss, and increased intervertebral height. The UBED group had a longer incision length, operation time and postoperative hospital stay, and fewer intraoperative fluoroscopy times than to the PEID group. Complications were noted in both groups throughout the follow-up period, but there was no significant difference in the rate of complications. Moreover, there were no notable differences in clinical outcomes between the two groups in the high-grade migrated LDH.

**Conclusions:**

Both UBED and PEID could achieve favorable clinical outcomes for treating migrated LDH at the lower lumbar spine. Despite the longer operative time and postoperative hospital stay associated with the UBED group, UBED remains safe and innovative for treating migrated LDH at the lower lumbar spine.

## Background

Lumbar disc herniation (LDH) is marked by the degeneration of the intervertebral disc in the lumbar region, rupture of the fibrous ring, herniation of the nucleus pulposus tissue, and resulting compression and irritation of nerve roots, leading to symptoms such as lower back pain and limb numbness [1].Migrated LDH represents a distinct subset of LDH, where the annulus fibrosus is completely ruptured, allowing the nucleus pulposus to pass through the posterior longitudinal ligament and into the spinal canal [2]. This type of LDH usually manifests with severe clinical symptoms that are not effectively managed with conservative treatment, often necessitating surgical intervention [3]. Advancements in minimally invasive technology have provided orthopedic surgeons with unconventional alternatives in the form of spinal endoscopic procedures for treating migrated LDH. Percutaneous endoscopic lumbar discectomy (PELD) involves minimal paravertebral muscle dissection and has evolved into a sophisticated technique for minimally invasive treatment of various types of LDH [4–6]. UBED is distinct from the coaxial mode of intervertebral foraminal endoscopy in that its observation and operation channels are separate and function without overlap [7–10].

The lower lumbar spine, consisting of the L4 and L5 segments, is characterized by an expanded interlaminar gap, allowing endoscopic access using a single or dual-channel interlaminar approach [11]. This anatomical feature provides sufficient space for maneuvering. It enables clearer visualization and precise manipulation of the herniated disc during the surgical procedure, reducing surgical trauma and improving safety. Percutaneous endoscopic interlaminar discectomy (PEID), as a model of single-port endoscopic surgery, combines the advantages of both microscopic and interlaminar approaches, and has achieved excellent results, especially in the treatment of LDH in the lower lumbar spine [12]. Similarly, the key characteristic of UBED lies in its non-interference between the two channels of observation and operation, providing it with the benefits of both open and conventional minimally invasive techniques [7, 13]. With the ability to maintain a clear field of view and precise instrument manipulation, UBED offers advantages in addressing complex and migrated LDH cases, minimizing the risk of nerve injury, and achieving favorable patient outcomes. Prior research has indicated that both UBED and PEID have resulted in favorable clinical outcomes in the management of LDH [7–9, 12]. However, few studies compared the efficacy of these two endoscopic discectomy techniques in treating migrated LDH. Therefore, the purpose of this study is to comprehensively compare the short-term efficacy of UBED and PEID in treating migrated LDH in the lower lumbar spine, exceptionally high-grade migrated LDH.

## Material and methods

### Patients and study design

This study was approved by our Institutional Review Board. Since the study involved reviewing existing data, informed consent was waived. A retrospective analysis was conducted on a cohort of patients who were diagnosed with migrated LDH of the lower lumbar spine and underwent UBED or PEID at a single hospital from January 2021 to January 2023. To minimize variation in clinical outcomes due to surgical experience and expertise, all procedures were performed by a single surgeon who had at least two years of experience in PEID surgery prior to January 2021 and had performed more than 50 UBE procedures.

All patients underwent routine preoperative lumbar spine radiography (lateral and dynamic views), computed tomography (CT), and magnetic resonance imaging (MRI). Inclusion criteria for the study were as follows: (1) Fulfillment of the diagnostic criteria for LDH. (2) MRI examination confirmed L4/5 or L5/S1 single-segment LDH. (3) Radiating pain and/or numbness in one or both lower limbs with a positive straight leg raising test, accompanied by varying degrees of low back pain. (4) Non-invasive treatments are ineffective, or the condition has worsened to a degree that significantly affects quality of life. Exclusion criteria were as follows: (1) Patients with lumbar spine tumors or infectious diseases. (2) Lumbar spondylolisthesis, lumbar instability, lumbar deformity, and severe lumbar laminitis. (3) History of lumbar surgery. (4) Inability to tolerate surgery due to other serious illnesses.

**Fig. 1:**
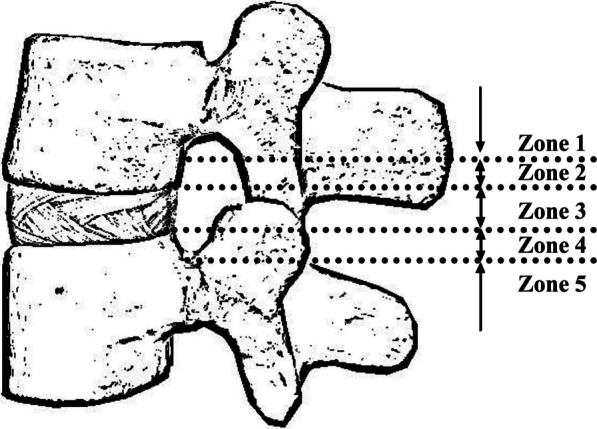
Schematic diagram of the radiological divisions of a herniated disc. The herniated discs are categorized into five regions based on the direction and degree of migration

Based on the preoperative MRI findings, Kang et al. [7] categorized disc herniation into five zones. These zones were determined based on the distance and direction from the disc space, resulting in the formation of five distinct groups: high-grade upward, low-grade upward, disc level, high-grade downward, and low-grade downward (Table 1; Fig. 1). Ultimately, we classified high-grade migration as Zones 1 and 5, and low-grade migration as Zones 2 and 4. Zone 3 was excluded from the study and designated as the disc level group. We hypothesized that comparable clinical outcomes could be achieved between UBED and PEID to treat migrated LDH. Furthermore, we further analyzed the outcomes of both techniques, explicitly focusing on high-grade migrated LDH.

**Table 1:** Radiographic zoning of disc herniation

Zone	Location	Range of distance
Zone 1	High-grade up	Above the lower half of the inferior pedicle
Zone 2	Low-grade up	Between the upper margin of disc space and the upper half of the inferior pedicle,
Zone 3	Disc level	From the inferior margin of upper vertebral body to the superior margin of lower vertebral body
Zone 4	Low-grade down	From the superior margin of lower vertebral body to the center of lower pedicle
Zone 5	High-grade down	From the center to the inferior margin of lower pedicle

### Surgical technique

**UBED Group** All patients received general anesthesia with endotracheal intubation and were subsequently positioned prone with soft pads supporting their face and abdomen. To locate the targeted intervertebral space under fluoroscopy, a horizontal line was marked at the junction of the spinous process and the lower edge of the vertebral plate, followed by a vertical line along the inner edge of the pedicle on the access side. Two skin incisions were created 1.5 cm cephalad and caudad from the intersection of the two lines. We created two portals by making longitudinal incisions into the lumbar spine and dorsal fascia, enlarging and bluntly separating the soft tissues covering the bony surfaces of the vertebral plate layer by layer to create an observation portal and a working portal. The arthroscopic system was inserted into the observation portals, and saline irrigation was used under hydraulic pressure to achieve minimal bleeding from tiny intra-spinal canal veins while maintaining a clear field of vision. Smooth water flow was essential to ensure clear operative visualization during UBE. The intervertebral soft tissue was treated and hemostasis was achieved through a plasma electrocoagulation system. The arthroscopic dynamic power drill and vertebral plate bite forceps were then utilized to remove a segment of the superior articular eminence and inferior vertebral plate, as well as the exposed ligamentum flavum and excise the ligamentum flavum. Retract and preserve the dura mater towards the midline to expose the nerve root and protruding nucleus pulposus tissue, then extract the loose nucleus pulposus tissue using nucleus pulposus forceps. Probe that the nerve root compression was relieved, the nerve root canal was relaxed, and then the intervertebral space was flushed. Seeing that the dural sac was beating well, the instruments and endoscope were withdrawn, and the incision was sutured.

**PEID Group** PEID was conducted with the patient under general anesthesia and positioned in a prone position. An anteroposterior fluoroscopy was done to locate the entry point landmark at the lateral edge of the interlaminar space. Following a 1 cm incision, the tandem dilator was introduced and positioned at the interslice space, distinct from creating a workspace for UBED. The working channel was introduced and final positioning was examined via fluoroscopy. An endoscopic system was introduced through a radiofrequency probe to expose the ligamentum flavum. Due to the restricted working tube, special instruments were utilized for single-port endoscopy, which differed substantially from those used in UBE. A portion of the ligamentum flavum was removed to uncover the nerve root while safeguarding it with a tube to reveal the intervertebral disc. Subsequently, the herniated or isolated disc is removed to alleviate pressure on the nerve root.

During the UBED procedures, drainage tubes were provided to patients who experienced bleeding (more than 50 ml) due to bony structures blocking the view that require bone grinding. Subsequently, the drainage tubes were removed once the drainage volume was less than 30 ml within 24 h. Both groups received postoperative anti-inflammatory, analgesic, and rehydration therapy for 1–2 days. On the first postoperative day, patients were permitted to wear lumbar support braces and engage in supervised ambulation while being cautioned against excessive lower back bending and strenuous activity. Over the ensuing three weeks, patients were primarily confined to bed and gradually escalated their physical activity until reaching preoperative levels by the end of the three weeks.

**Fig. 2:**
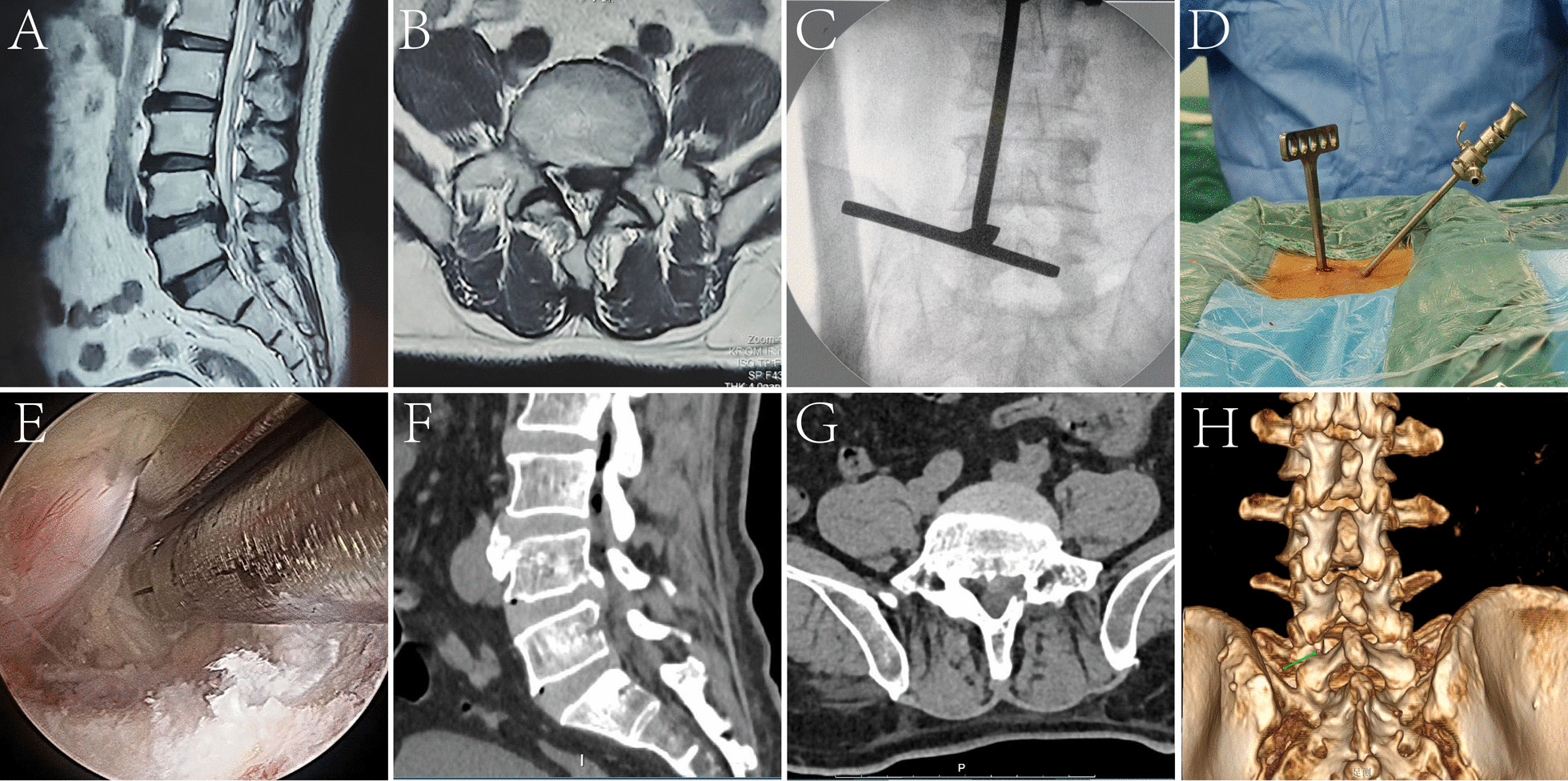
Surgical procedure of UBED and pre/postoperative imaging of patients with high-grade downward migrated LDH. **A**, **B** Preoperative sagittal and transverse MRI showed high-grade downward migration of the L4/5 disc. **C**–**E** Image C depicts the anteroposterior fluoroscopy utilized to guide the UBED procedure. Image D highlights the UBED working channel. Image E displays the decompressed nerve root following successful UBED. **F**–**H** Postoperative sagittal and coronal CT imaging of the operated area reveals the absence of any remaining migrated nucleus pulposus. Additionally, 3D reconstruction enables clear visualization of the defective bone

Typical Case 1: A 45-year-old female patient suffered from varying degrees of pain in both lower limbs and mild lower back pain. Preoperative MRI showed L4/5 intervertebral disc prolapse with high-grade downward migration. Following UBED treatment at our hospital, the patient experienced a significant reduction in leg pain (Fig. 2).

**Fig. 3:**
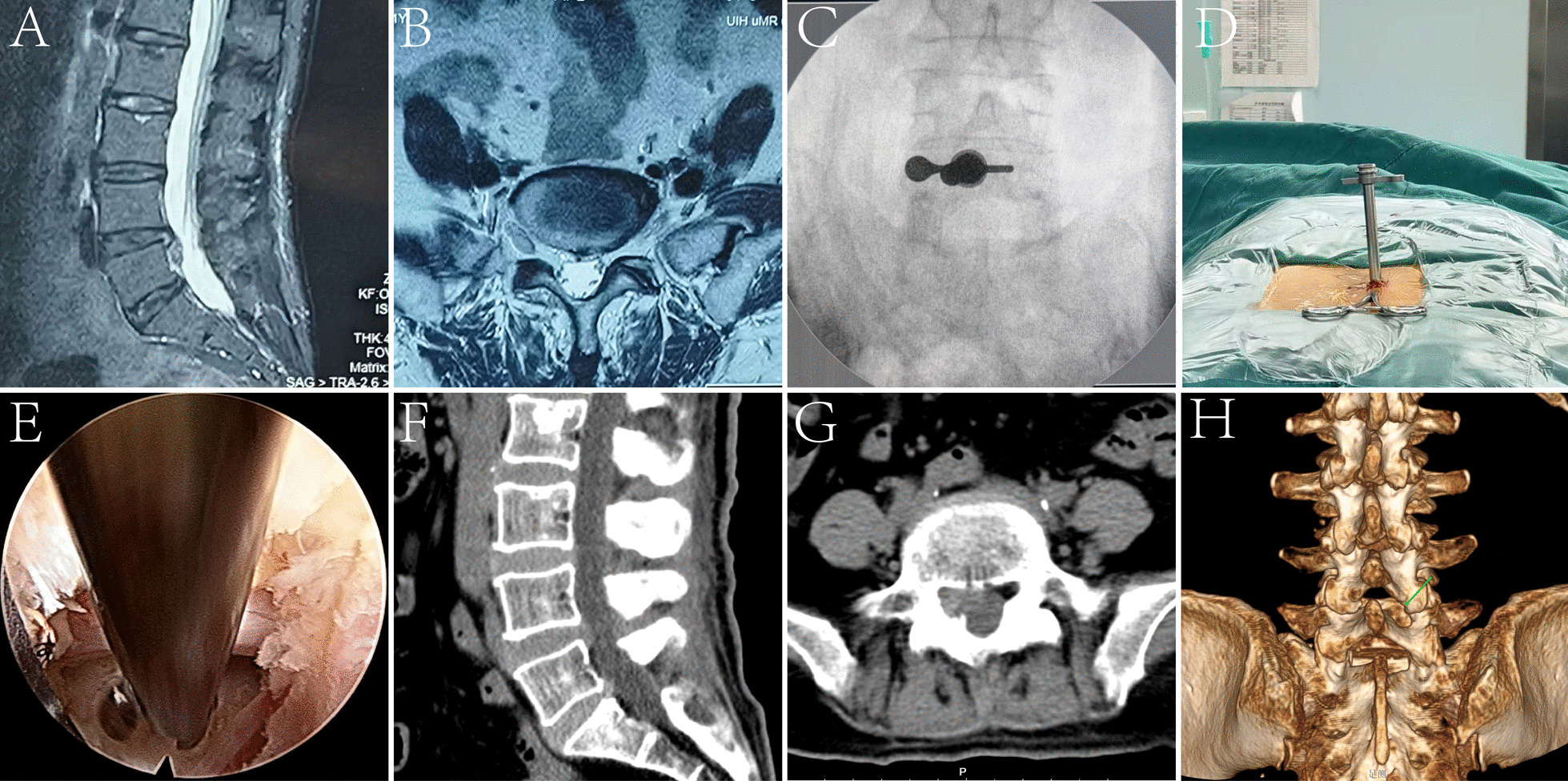
Surgical procedure of PEID and pre/postoperative imaging of patients with high-grade downward migrated LDH. **A**, **B** Preoperative sagittal and transverse MRI showed high-grade downward migration of the L4/5. **C**–**E** Image C showed the anteroposterior fluoroscopy utilized during the PEID procedure. Image D showed the PEID working channel. Image E demonstrated the decompressed nerve root following PEID. **F**–**H** Postoperative sagittal and coronal CT imaging of the operated area reveals the absence of any remaining migrated nucleus pulposus. Additionally, 3D reconstruction enables clear visualization of the defective bone

Typical Case 2: A 50-year-old female patient presented with chronic lower back pain and left lower limb radiation persisting for over a year. Preoperative MRI showed L5/S1 intervertebral disc prolapse with high-grade downward migration. Following the PEID procedure at our hospital, the patient exhibited a notable decrease in both lower back and lower limb pain. Subsequent postoperative CT imaging demonstrated the absence of residual nucleus pulposus in the operated segment. (Fig. 3).

### Observation index

Perioperative indicators: operation time (time from skin incision to suturing), hemoglobin loss (hemoglobin concentration on the day before operation minus hemoglobin concentration on the day after operation), fluoroscopy times, total incision length, and postoperative hospital stay.

Efficacy indicators: Visual Analog Scale (VAS) scores for lower back and leg pain and Oswestry Disability Index (ODI) were collected preoperatively, postoperative first day, 1-month, 6-months, and last follow-up. Modified MacNab criteria were collected at 6 months, and surgery-related complications were recorded during follow-up.

**Fig. 4:**
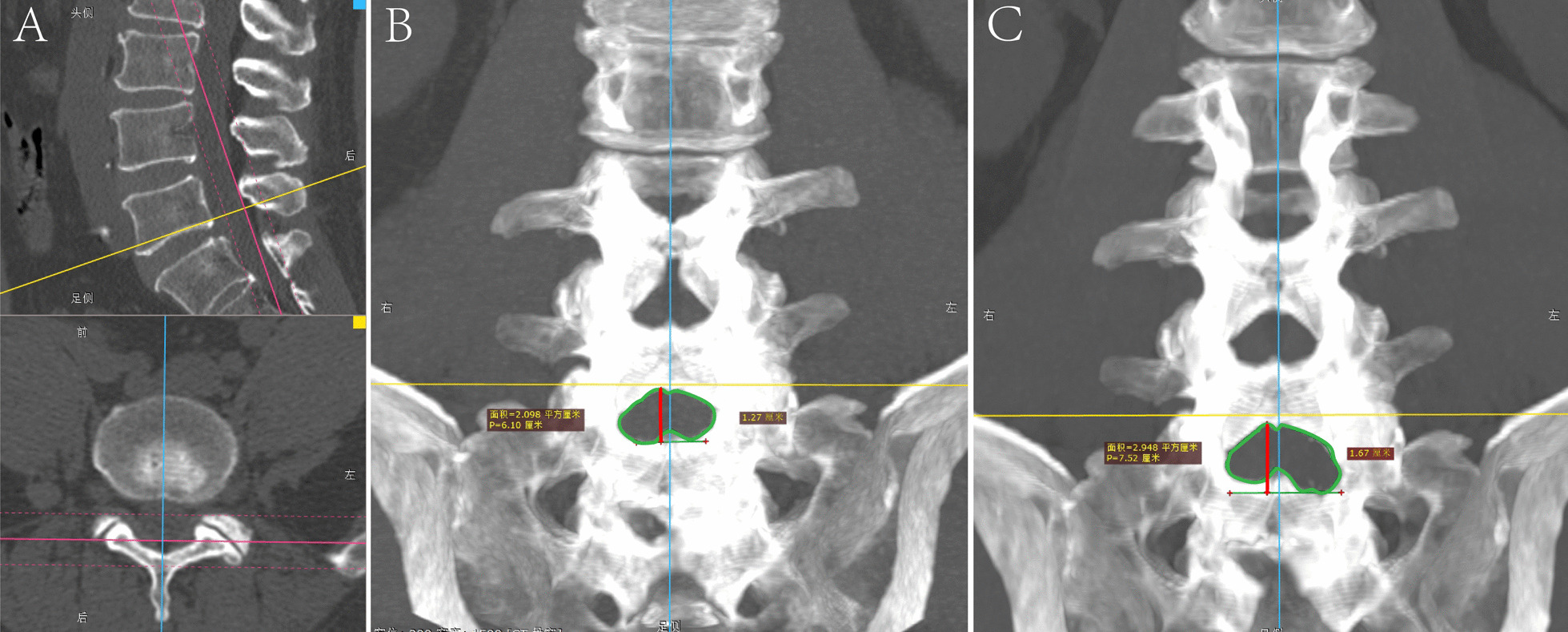
Measurement of lamina loss area and height changes. **A** The MIP plane was parallel to the longitudinal axis of the target vertebral body in the sagittal position, and the distance in the transverse position was from the facet joint to the junction of the spinous process and the lamina. **B**, **C** Significant differences in the area and height of the interlaminar space were shown by the maximum density projections before and after surgery

Indicators of the intervertebral space: Thin-layer CT data were loaded with RadiAnt DICOM Viewer software (Version 2021.2, Medixant, Poland). All key parameters were measured on CT in bone mode. Maximum Intensity Projection (MIP) was employed to visualize the dimensions of the interlaminar space in terms of area and height. The selected layer thickness was the distance from the facet joint to the junction of the spinous process and the lamina in the transverse position and was parallel to the longitudinal axis of the target vertebral body in the sagittal position. (Fig. 4) The area of lamina loss was defined as the difference between the postoperative and postoperative lamina space areas. The height of the intervertebral space was defined as the vertical distance between the highest and lowest points of the intervertebral space. Two orthopedic spine surgeons independently measured each parameter three times, and the final dataset was derived from the average of these six measurements.

### Statistical analysis

Quantitative data were expressed as mean ± standard deviation, and all statistical data were analyzed by Statistical Package for the Social Sciences (SPSS) 27.0 software (SPSS, Inc., Chicago, IL, USA). T-test was used for measurement data, and intergroup comparison and counting data used χ^2^ inspection Fisher's exact probability method. Comparisons with values of *P* < 0.05 were considered statistically significant.

## Results

A total of 96 patients, including 31 cases in the UBED group and 65 cases in the PEID group, met the inclusion criteria. No significant differences were observed between the two groups regarding age, gender, BMI, disc level, disc location, and duration of follow-up. (Table 2).

**Table 2:** The demographic data in UBED and PEID

	UBED (*n* = 31)	PEID (*n* = 65)	*P*
Age (years)	58.5 ± 13.2	57.6 ± 16.8	0.780
BMI (kg/m2)	24.1 ± 3.0	25.0 ± 2.9	0.155
Gender			0.521
Male	16	29	
Femal	15	36	
Disc level			0.927
L4/5	17	35	
L5/S1	14	30	
Disc location			0.846
Low-grade	23	47	
High-grade	8	18	
Follow-up duration (months)	8.6 ± 2.9	12.3 ± 4.6	< 0.01

No significant differences between the two groups were observed in the preoperative VAS scores and ODI (*p* > 0.05). Postoperatively, both groups exhibited significantly improved VAS scores and ODI (*p* < 0.05). Furthermore, there were no significant differences in the VAS scores and ODI between the two groups on the first day, 1-month, 6-month, and final follow-up after surgery (*p* > 0.05). In addition, there were no significant differences in the preoperative and postoperative areas of lamina loss and height changes between L4/5 and L5/S1. The UBED group exhibited significantly longer incision length, operation time and postoperative hospital stay compared to the PEID group (*P* < 0.05). Conversely, the intraoperative fluoroscopy times for the UBED group were significantly lower than those for the PEID group (*P* < 0.05). However, there was no significant difference in hemoglobin loss (*P* > 0.05). Additionally, there was no significant difference in both excellent/good rate (90.3% vs. 84.6%) and postoperative complications (3.2% vs. 3.1%) between the two groups (*P* > 0.05). (Tables 3 and 4).

**Table 3:** Comparison of perioperative conditions between the UBED and PEID

	UBED (*n* = 31)	PEID (*n* = 65)	*P*
Operation time (min)	77.3 ± 20.4	65.8 ± 15.9	0.003
Hb loss (g/L)	8.3 ± 5.0	7.7 ± 3.3	0.503
Intraoperative fluoroscopy times (n)	3.4 ± 0.9	4.8 ± 1.0	< 0.001
Incision length (mm)	23.9 ± 3.5	16.4 ± 2.5	< 0.001
Postoperative Hospital stay (days)	4.5 ± 2.6	3.1 ± 1.8	0.004

**Table 4:** Clinical outcomes of UBED and PEID

	UBED (*n* = 31)	PEID (*n* = 65)	*P*
VAS back			
Preoperative	5.2 ± 1.1	5.4 ± 1.1	0.343
1 day after operation	2.5 ± 0.6	2.5 ± 0.6	0.670
1 month after operation	2.1 ± 0.8	2.0 ± 0.6	0.602
6 months after operation	1.6 ± 0.7	1.7 ± 0.6	0.431
Last follow-up	1.5 ± 0.5	1.5 ± 0.5	0.931
VAS leg			
Preoperative	6.8 ± 0.9	6.6 ± 0.9	0.263
1 day after operation	2.6 ± 0.6	2.4 ± 0.8	0.141
1 month after operation	2.0 ± 0.7	2.1 ± 0.8	0.783
6 months after operation	1.8 ± 0.7	1.9 ± 0.9	0.582
Last follow-up	1.5 ± 0.5	1.8 ± 0.8	0.102
ODI			
Preoperative	50.5 ± 7.0	51.3 ± 10.4	0.698
1 day after operation	21.1 ± 4.6	19.8 ± 4.2	0.176
1 month after operation	7.6 ± 2.2	8.6 ± 2.6	0.085
6 months after operation	5.9 ± 2.3	6.2 ± 2.5	0.637
Last follow-up	5.7 ± 1.9	5.8 ± 2.3	0.876
Area of lamina loss (mm^2^)			
L4/5	82.3 ± 26.6	79.7 ± 26.2	0.740
L5/S1	59.1 ± 19.5	51.1 ± 22.7	0.258
Increased intervertebral height (mm)			
L4/5	3.4 ± 1.8	2.6 ± 1.4	0.058
L5/S1	2.4 ± 1.6	3.0 ± 1.8	0.362
MacNab			
(excellent/good/fair/poor)	16/12/1/2	36/18/8/3	0.917
Excellent/good rate	90.3%	84.6%	0.656
Complications, n (%)	1(3.2%)	2(3.1%)	1.000

The efficacy of patients with high-grade migrated LDH in the UBED and the PEID groups was also contrasted. No statistically significant disparities were evident between the two groups in terms of postoperative VAS scores, ODI, and MacNab (*P* > 0.05). Likewise, there was no significant difference in the excellent/good rates or postoperative complications within either group (*P* > 0.05). (Table 5).

**Table 5:** Clinical outcomes of UBED and PEID in the treatment of high-grade migrated LDH

	UBED (*n* = 31)	PEID (*n* = 65)	*P*
VAS back			
Preoperative	5.6 ± 1.1	5.5 ± 1.2	0.808
1 day after operation	2.4 ± 0.7	2.4 ± 0.5	0.956
1 month after operation	2.3 ± 0.7	2.1 ± 0.7	0.531
6 months after operation	1.9 ± 1.0	1.6 ± 0.5	0.371
VAS leg			
Preoperative	6.8 ± 0.9	6.6 ± 0.8	0.872
1 day after operation	2.6 ± 0.5	2.7 ± 1.1	0.252
1 month after operation	2.3 ± 0.7	2.2 ± 0.9	0.325
6 months after operation	1.5 ± 0.5	2.0 ± 1.0	0.241
ODI			
Preoperative	52.3 ± 7.6	48.3 ± 6.8	0.425
1 day after operation	21.8 ± 3.5	18.6 ± 4.5	0.490
1 month after operation	7.5 ± 2.3	8.9 ± 1.7	0.145
6 months after operation	6.8 ± 2.1	5.9 ± 2.5	0.790
MacNab			
(excellent/good/fair/poor)	5/1/1/1	8/4/5/1	0.691
Excellent/good rate	75.0%	66.7%	0.656
Complications, n (%)	0(/)	2(3.1%)	1.000

Three surgical complications were observed during the follow-up period. The UBED group reported one case of dural tear, while the PEID group experienced one case of dural tear and one incidence of nerve root injury.

## Discussion

At the 1-day, 1-month, and 6-month follow-ups, both groups demonstrated significant improvements in pain scores and functional status. As assessed by the modified MacNab criteria, patient satisfaction suggested comparable efficacy of the UBED and PEID techniques in managing migrated LDH. However, the UBED technique was associated with notably longer incisions, extended operation times, prolonged postoperative hospital stays, and augmented utilization of intraoperative fluoroscopy.

During the UBED procedure, the observation and the operation channels remain distinct and do not disrupt each other. The endoscope in the observation channel possesses a narrower diameter, enabling adjustable angle and positioning. Additionally, the decompression instrumentation can flexibly explore and accomplish decompression in all directions of the spinal canal, providing an even greater advantage for the nucleus pulposus migrating upward or downward [7, 13, 14]. PEID is implemented through a single portal in the posterior interlaminar approach, thereby avoiding obstruction of structures such as the lateral pedicles, superior articular eminences, and high iliac crests, compared to the intervertebral foraminal approach [15–17]. As a result, it surpasses PELD in treating migrated disc herniation [16]. The anatomical trajectory and endoscopic perspective of UBED and PEID closely mirror those of traditional posterior laminectomy and discectomy. Both procedures require the removal of a segment of the lamina after gradual stripping of the muscle layers to establish a pathway between the intervertebral spaces. Abundant research has revealed that both interventions can yield favorable clinical results for individuals with lumbar disc herniation, particularly in the lower lumbar spine [11, 12, 17]. In this study, both UBED and PEID demonstrated favorable clinical outcomes regarding VAS and ODI scores in the immediate postoperative period and during the follow-up period. No significant difference was observed between the two groups.

The present study shows that the UBE group had a longer operation time and postoperative hospital stay than that of the PEID group, partially aligning with previous research findings. According to Choi et al. [18], the mean operative time was 96.15 ± 16.97 min in the UBED group and 85.52 ± 17.79 min in the PEID group, which is a significant difference (*p* < 0.05). Heo et al. [19] observed that the mean duration for the UBED group was 62.4 ± 5.7 min, while the PELD was timed at 61.6 ± 3.0 min. As a result, the PEID group exhibited a significantly shorter duration of surgery and postoperative hospitalization compared to the UBED group. As a result, the PEID group exhibited a significantly shorter duration of surgery and postoperative hospitalization compared to the UBED group. This outcome can likely be attributed to the procedural requirement of the UBED technique to sequentially create two channels during the surgical procedure. Consequently, this extended the surgical duration and increased muscle damage in the paravertebral muscles, especially the erector spinae muscle. As a result, the recovery process postoperatively was delayed, necessitating longer hospital stays. In this study, the lead surgeon had extensive experience with PEID surgery and was well versed in lumbar spine anatomy, so the learning curve for UBED was minimally challenging for him. A study by Xu et al. [20], involving 197 patients who underwent UBED surgery, noted that surgeons only began to observe a reduction in operative time and postoperative hospital stay after performing 32 UBED cases. The surgeon who performed our UBED procedure had experience with more than 50 cases of UBED prior to the start of the study, so clinical outcomes were minimally affected by the learning curve of UBED.

UBED and PEID procedures involve removing a portion of the lamina or facet joint to create sufficient room for the channel placement. Long-term bone loss negatively affects the patient's spinal stability [21]. The increase in the intervertebral space before and after surgery can be a sign of lamina bone loss. X-ray imaging was employed in early studies to quantify lumbar intervertebral parameters [22]. MIP is a post-processing method for CT 3D reconstruction that projects voxels with the highest CT values onto a background plane with a certain thickness (CT slice thickness) to display concentrated, dense vessels and organs. MIP is primarily used for the diagnosis of pulmonary nodules and to show vascular alignment [23, 24]. In our study, MIP was applied to measure interlaminar space area. The superiority of MIP over conventional X-ray measurements of the intervertebral space lies in its ability to more accurately display the boundaries of the intervertebral space and selectively exclude the influence of other levels on area measurements. In our study, no significant differences were observed in the enlargement of laminar space area and height changes between the UBED and PEID groups for either L4/5 or L5/S1 segments. This suggests a slight disparity between the two surgical approaches regarding the excised bone tissue during the creation of channels.

One case of dural tear was observed in the UBED and PEID groups. Previous research has established dural tears as the most common complication of UBED [25, 26]. Lin et al. [25] conducted a comprehensive study that included statistical analysis of six studies, which concluded that the average incidence of postoperative dural tears following UBED was 4.1%. Risk factors for dural tears encompass spinal canal adhesion, dura mater damage caused by instruments or radiofrequency, dura loosening, and substantial disc fragments. Therefore, delicately dissecting the meningo-vertebral ligament is particularly important [27]. It is recommended to observe simple absolute bed rest for small tears of less than 4 mm, while larger defects with a size greater than 10 mm can be remedied via an open restoration, which is considered to be a safe technique [28]. Nevertheless, further refinement of the dural endoscopy repair technique is required to enhance both patient safety and procedural efficacy.

Radiation exposure during endoscopic spinal surgery is a prominent threat for surgeons in contemporary practice. The deleterious impact of radiation is observed on both patients and surgeons, thus emphasizing the significance of minimizing exposure. A study by Merter et al. [29] compared and analyzed three different endoscopic discectomies for treating LDH in terms of intraoperative radiation exposure and found that the ranking was PELD > UBE > MED based on the duration and level of radiation exposure. In this study, we analyzed the impact of PEID versus UBED on intraoperative radiation exposure with respect to the frequency of intraoperative fluoroscopy procedures. Our findings demonstrated a significantly higher number of fluoroscopic sessions associated with the use of PEID compared to UBED (*P* < 0.05), aligning with the outcomes of prior investigations.

There are limitations to this study. First, it was a retrospective study conducted in a single center with a small number of cases. Then, the UBED group had a mean follow-up of only six months, which is insufficient to assess clinical outcomes such as LDH recurrence and spinal instability. Therefore, future studies should include large-scale prospective multicenter trials comparing the long-term clinical outcomes of single-port endoscopic surgery with other microendoscopic procedures. It is important to use consistent sample sizes to ensure accurate and reliable results.

## Conclusion

As for the management of migrated LDH at the lower lumbar spine, both UBED and PEID can achieve comparable favorable clinical outcomes, including pain relief, patient satisfaction, preservation of posterior bony structures, and complications. This may be attributed to the steep learning curve associated with this innovative technique despite requiring longer operative time and postoperative hospital stay for the UBED group. In summary, UBED may serve as a safe and innovative alternative for treating migrated LDH at the lower lumbar spine. However, a significant learning curve must be traversed to minimize the incidence of adverse effects.
